# Early lateral migration of head after bipolar hemiarthoplasty in a cerebral palsy patient

**DOI:** 10.4103/0019-5413.69321

**Published:** 2010

**Authors:** Ken Hiragami, Arata Mukohyama, Yoshiyuki Maruyama

**Affiliations:** Department of Orthopaedic Surgery, Saka General Hospital, Miyagi, Japan; 1 Department of Orthopaedic Surgery, Tachikawasougo General Hospital, Tokyo, Japan

**Keywords:** Cerebral palsy, femoral neck fracture, hemiarthroplasty, migration

## Abstract

Migration of the outer head after bipolar hemiarthroplasty within several years after surgery is not a rare complication. We present a patient with cerebral palsy who showed lateral migration of the outer head seven months after bipolar hemiarthroplasty for femoral neck fracture. The patient had no acetabular pathology prior to the fracture, and lacked ambulatory ability in a community setting. She underwent conversion to a total hip arthroplasty and returned to her previous lifestyle.

## INTRODUCTION

The treatment of displaced femoral neck fractures in elderly patients is controversial. Although the outcomes of total hip arthroplasty are superior to those of hemiarthroplasty,^1^ some authors advocate that hemiarthroplasty has a good outcome in the elderly if acetabular disease is absent.^2^,^3^ Bipolar hemiarthroplasty tends to be indicated for sedentary patients because of the possibility of migration of the outer head of bipolar.^4^ We report a patient with cerebral palsy in whom migration of the outer head occurred seven months after bipolar hemiarthroplasty for a femoral neck fracture. She had no acetabular disease before the fracture and lacked ambulatory ability in a community setting.

## CASE REPORT

A 60-year-old woman with athetoid cerebral palsy suffered a displaced femoral neck fracture of the left hip in 2004. She had been able to transfer from the bed to the wheelchair independently before the injury, but she lacked ambulatory ability in a community setting.

**Figure 1:**
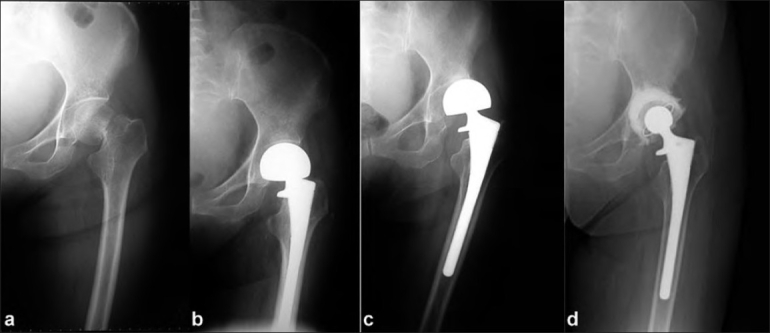
(a) An anteroposterior radiograph of left hip showing a femoral neck fracture and a radiologically normal acetabulum at the time of the injury (b) Immediate postoperative anteroposterior radiograph of the left hip showing no subluxation, (c) 9 months postoperative anteroposterior radiograph showing lateral migration of outer head. (d) Two years postoperative anteroposterior radiograph after conversion to total hip arthroplasty showing no signs of loosening or change in the position of the implant

Radiography revealed that the sharp angle^5^ of the left hip was 40° at injury [Figure 1a]. The patient had no acetabular pathology prior to fracture. Five months later, she underwent bipolar hemiarthroplasty at another hospital and recovered uneventfully [Figure 1b]. She had been able to return to her previous lifestyle, but she presented to us with a two-month history of severe pain in the left groin nine months after the hemiarthroplasty. Pain was aggravated by athetoid movement and increased muscle tone even at rest. Non-steroidal anti-inflammatory drugs did not relieve the pain.

At the first visit to our hospital, standard radiographs of the left hip demonstrated lateral migration of the prosthetic bipolar head [Figure 1c]. Laboratory examinations revealed a normal C-reactive protein level and white blood cell count. Physical examination revealed mild restriction of left hip motion due to pain. She tended to adduct and extend her left hip with increased muscle tone. Antispasmodic drugs (p.o.) had little effect on pain.

Four weeks later, in view of the severe continuous pain, we removed the outer head and placed the acetabular component with cement and without a bone graft. Intraoperatively, the lateral aspect of the acetabulum was found to be denuded of cartilage. We concomitantly performed release of the adductor muscles and medial hamstrings. A sample of clear synovial fluid taken from the joint at the time of surgery did not produce bacterial growth on culture.

At two years follow-up after the conversion, she could transfer independently between the bed and wheelchair with mild pain in the left groin, but the pain subsided at rest even with increased muscle tone. A plain radiograph revealed no signs of loosening or change in the position of the implants [Figure 1d].

## DISCUSSION

Some reports have described fracture of the femoral neck in patients with cerebral palsy. Ries reported recurrent dislocation of the hip after hemiarthroplasty in two patients with cerebral palsy who suffered femoral neck fracture and underwent posterior allograft bone-block.^6^ In 1999, Weber *et al.* reported total hip arthroplasty in 16 patients with cerebral palsy, including conversion from endoprosthesis failure after femoral neck fracture.^7^ However, they did not indicate if migration of the outer head had occurred. Consequently, the outcome of hemiarthroplasty for cerebral palsy patients suffering femoral neck fracture has remained unclear.

Authors have reported that total hip arthroplasty is a valuable option for the treatment of painful osteoarthritis in patients with cerebral palsy, and that the long-term outcome is favorable for both severely disabled and ambulant patients.^7^ Buly *et al*. reviewed 19 hips in patients with cerebral palsy and found that the 10-year survival rate was 95%.^8^

Hip problems are common in children with cerebral palsy; the reported prevalence of hip dislocation being 6.5–59%.^9^,^10^ The most common primary factor has been considered to be muscle imbalance, with adductors, flexors and medial hamstrings being relatively overactive compared with their antagonists.^11^

In our patient, hemiarthroplasty was not considered a contraindication in view of the lack of data on the outcome of treatment for femoral neck fracture in cerebral palsy patients, the patient’s advanced age, her limited mobility and the absence of significant acetabulum disease.

Because there was no evidence of infection, the most likely cause for early migration of the outer head was abnormal muscle tone. Release of the long adductor muscle and medial hamstring was performed in addition to conversion to total hip arthroplasty because of her tendency to extend and adduct her left hip with increased muscle tone.

Because of a few previous reports, we hope that this report will encourage the reporting of similar cases and lead to better treatment of this condition.
